# Inter-Trial Variability of Context Influences the Binding Structure in a Stimulus-Response Episode

**DOI:** 10.5334/joc.215

**Published:** 2022-04-07

**Authors:** Ruyi Qiu, Malte Möller, Iring Koch, Susanne Mayr

**Affiliations:** 1 Chair of Psychology and Human-Machine Interaction, University of Passau, 94032, Passau, Germany; 2 Department of Cognitive and Experimental Psychology, RWTH Aachen University, 52056, Aachen, Germany

**Keywords:** context, inter-trial variability, stimulus-response episode, binding

## Abstract

There is strong evidence that stimuli and responses are bound together in a direct (*binary*) fashion into an episodic representation called *stimulus-response episode* (or *event file*). However, in an auditory negative priming study in which participants were required to respond to the target stimulus and to ignore the distractor stimulus, context information (i.e., a completely task-irrelevant stimulus) was found to rather modulate the binding between the distractor stimulus and the response, instead of entering into a binary binding with the response itself (Mayr et al., 2018). The current study demonstrates that simply increasing the variability of the context across trials leads to a binary binding between the context and the response. The same auditory negative priming task was implemented, and participants were either assigned to the high-variability group (8 different context sounds) or the low-variability group (2 different context sounds). For the low-variability group, results replicated previous findings of contextual modulation of the binding between the distractor stimulus and the response. For the high-variability group, however, repetition of the context per se retrieved the prime response, indicating a binary binding between the context and the response. Together, the current findings provide evidence that the inter-trial variability of context information is a determinant of how context is bound in a stimulus-response episode. Possible underlying mechanisms are discussed.

## Introduction

The human brain encodes different perceptual features (e.g., color, shape, pitch, and loudness) of stimuli in a distributed fashion (e.g., Seymour et al., 2009; Stecker et al., 2005). To achieve a coherent perception of the world requires mechanisms that bind features (e.g., Kahneman et al., 1992; Treisman, 1996). Apart from the perceptual features, a multitude of findings suggests that action features (e.g., a keypress response) are bound as well (for an overview of feature binding across perception and action, please see Hommel, 2004). It is assumed that the stimulus and the response can be integrated into a common episodic representation, referred to as a stimulus-response (S-R) episode (or an event file). Upon reencountering a feature stored in an S-R episode, the whole episode is retrieved, which either facilitates or impairs performance, depending on whether the retrieved information is compatible with the current processing requirements or not. The so-called *S-R binding and retrieval* processes have been proved to be a common mechanism underlying human information processing and action control (for a recent review of the binding and retrieval in action control, *BRAC*, please see Frings et al., 2020). Previous findings further indicate that the binding process usually results in a so-called binary structure, which links the individual stimulus and the response (e.g., Hommel, 1998, 2004; Moeller et al., 2016; Singh & Frings, 2020).

**Figure 1:**
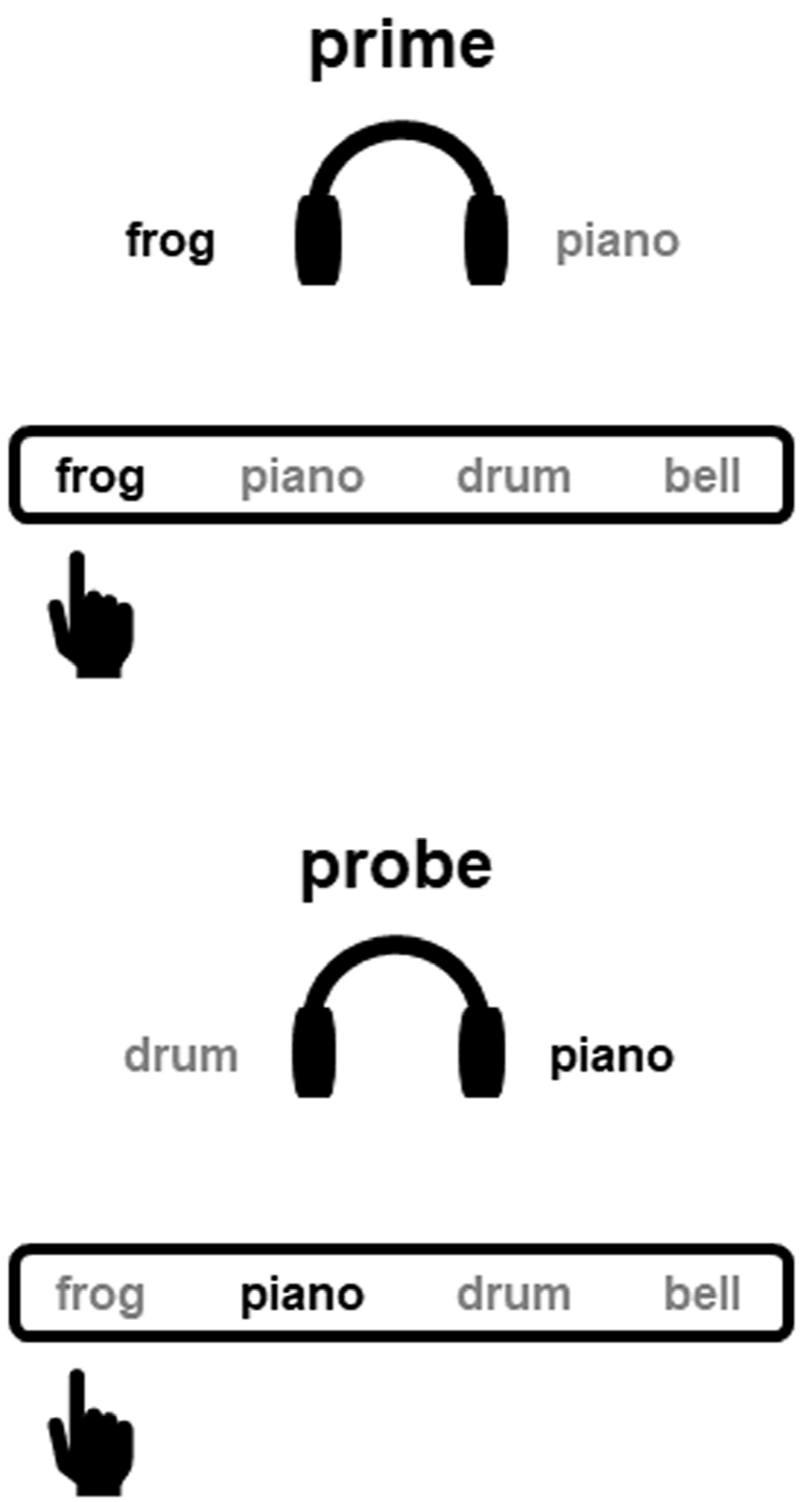
Illustration of the erroneous probe response with the previously executed prime response in ignored repetition trials. *Note*: Targets and correct responses are in black, distractors and incorrect responses are in grey.

Supplementing the relevance of S-R bindings in human action, the binary binding between a previously ignored stimulus and the executed response was found to be one of the causes underlying the *negative priming effect* (referred to as the prime-response retrieval account, Mayr & Buchner, 2006; for a similar account, please see Rothermund et al., 2005).^1^ Mayr and Buchner (2006) used an auditory identification task, in which participants were required to respond to a target sound via an appropriate keypress while ignoring a simultaneously presented distractor sound. Four environmental sounds were used as target and distractor stimuli, and each of the sounds was assigned to a unique response key. In the so-called *ignored repetition* trials, the distractor stimulus of the previous presentation (i.e., the *prime*) was used as the target in the following presentation (i.e., the *probe*). Probe reaction times were longer and probe error rates were higher in these ignored repetition trials than in trials devoid of any stimulus repetition (i.e., the *control* trials), resulting in the so-called negative priming effect. The further analysis of probe error frequencies showed that the participants were more likely to commit errors with the prime response in ignored repetition trials as compared with control trials. This finding reveals that the repetition of the prime distractor stimulus in the probe retrieves the executed prime response, indicating that these elements were bound together into the prime episodic representation (see ***Figure 1*** for illustration of the prime-response retrieval error). Since the retrieved prime response is incompatible with the required response in the probe, the emerging response conflict has to be overcome before a correct response can be given, contributing to the negative priming effect. The significant increase of prime response errors induced by the repetition of a stimulus has been coined the *prime-response retrieval effect*, which is considered as an unambiguous indicator of the (binary) binding between a stimulus and the response (Frings et al., 2015; Mayr et al., 2018).

Interestingly, in a recent study using the aforementioned four-alternative auditory negative priming task, an additional task-irrelevant stimulus that served as context was found to modulate the binding between the prime distractor stimulus and the response, instead of being bound in a binary fashion with the response itself (Mayr et al., 2018). A sine tone presented together with the target and distractor sounds was used as the context stimulus. The context tone could repeat or change between the prime and the probe. Results showed that the sole repetition of the context tone in the probe did not increase errors with the prime response (i.e., the prime-response retrieval effect induced by the sole repetition of the context was not significant). However, the prime-response retrieval effect induced by the repetition of the prime distractor stimulus was significantly larger when the context was *additionally* repeated than when it was changed. In other words, the context did not retrieve the prime response on its own, but increased the retrieval of the prime response induced by the repetition of the prime distractor stimulus. This finding cannot be explained by presumed binary bindings between stimuli and responses. Instead, it suggests that the context is involved in a higher-order binding (Hommel, 1998; Singh & Frings, 2020), which includes more than two elements (context, distractor, and response) in an episodic representation (Qiu et al., 2022). This novel finding leads to the questions whether context information can be bound in a binary fashion with the response as well, and if so, what determines the binding structure (i.e., binary vs. higher-order) involving context. The main purpose of the current study was to investigate if the inter-trial variability of context could influence whether it enters into a binary binding with the response or not. To this end, the four-alternative auditory negative priming task was employed as the vehicle to test the influence of context variability on the prime-response retrieval effect (induced by the repetition of the context and by the repetition of the prime distractor stimulus).

### The role of inter-trial contextual variability in negative priming

It is well-established in the memory literature that contextual information is encoded and the repetition of the encoded contextual information can improve successful retrieval (for a review, see Smith & Vela, 2001). Substantiating the contribution of memory-related processes, the negative priming effect was also found to be influenced by the manipulation of context repetition (e.g., Fox & De Fockert, 1998; Neill, 1997). Typically, a larger negative priming effect was found when the context was repeated than when it was changed, which is referred to as the *contextual similarity effect*. Chao (2009) investigated the contextual similarity effect with a focus on the inter-trial variability of context. A visual negative priming task was used, in which symbols (i.e., the context stimuli) and distractor letters flanked the central target letter (e.g., A @ B @ A). The symbols could repeat or change between the prime and the probe. In the low-variability condition, two different symbols served as the context, whereas sixteen different symbols were used in the high-variability condition. Results showed a significant negative priming effect in the high-variability condition when the prime context was repeated in the probe, but not when the context was changed. As for the low-variability condition, no significant negative priming effect was obtained, regardless of the context relation between the prime and the probe. An attention hypothesis was put forward by Chao (2009) for these results. It was assumed that more frequently changing context stimuli receive more attention during processing, ultimately leading to stronger encoding of the context information in memory (e.g., Chun & Turk-Browne, 2007; Logan, 1988; Uncapher et al., 2011). As a consequence, the context presumably acted as a more potent retrieval cue to the prime episode in the high- as compared with the low-variability condition, leading to the significant contextual similarity effect in the former. Additionally, according to the *cue overload* assumption (Watkins & Watkins, 1975), Chao (2009) also assumed that individual context stimuli in the low- as compared with the high-variability condition are associated with more (and different) representations. In this sense, context in the low-variability condition might be a less efficient retrieval cue of the most recent prime information as it is connected to several other episodes in memory as well. Since evidence suggests that it is the most recently formed S-R bindings that mainly matter in the current S-R retrieval process (e.g., Giesen et al., 2020; Schmidt et al., 2016), we suppose that cue overload may not play an important role in modulating the S-R binding and retrieval component of the negative priming effect, whereas attention can be a potential modulator of these episodic memory processes (Moeller & Frings, 2014).

In fact, only two context stimuli were used in the study by Mayr et al. (2018), resembling the low-variability condition employed by Chao (2009). However, a significant contextual modulation of the prime-response retrieval effect induced by the repetition of the prime distractor stimulus (i.e., the S-R binding and retrieval component of the negative priming effect) was found in Mayr et al. (2018). To reiterate, the prime-response retrieval effect was larger when the context was additionally repeated than when it was changed, but sole context repetitions did not increase the likelihood to perform the former prime response in the probe. Referring to the attention hypothesis by Chao (2009), it is possible that the context stimuli in Mayr et al. (2018) did not receive sufficient attention during processing to form a binary binding with the prime response. Instead, the context modulated the binding between the prime distractor stimulus and the response, which presumably is due to the fact that the context stimulus was less discriminable from other stimuli in the prime episode, thereby entering into a so-called configural binding with prime stimuli and the response (see Moeller et al., 2016 for evidence that discriminability determines binding structure).

### Current study

To test the attention-related hypothesis, in the present experiment, the identical four-alternative auditory negative priming task as in Mayr et al. (2018) was implemented, and a total of eight different sine tones were used as the context. Participants were randomly assigned to either the high-variability group (in which all of the eight sine tones were used as the context), or the low-variability group (in which two out of the eight sine tones were randomly selected as the context throughout the whole experiment). It was expected that in the high-variability group, the repetition of the context per se should retrieve the prime response (i.e., a significant prime-response retrieval effect induced by the repetition of the context should emerge), which indicates a binary binding between the context and the response. However, in the low-variability group, no significant prime-response retrieval effect induced by the repetition of the context per se should be found. Instead, the repetition of the context may promote the prime-response retrieval process induced by the repetition of the prime distractor stimulus, as shown in the previous study (Mayr et al., 2018). This would indicate the formation of a configural binding among context, distractor, and response.

## Method

### Participants

One hundred and three participants took part in the experiment, most of whom were students at the University of Passau. Eleven of these participants were tested offline, whereas the remaining ninety-two participants were tested online on the Pavlovia platform (*https://pavlovia.org*) due to the onset of the Covid-19 pandemic. Data of three participants had to be excluded because of excessive error rates (over 50%) in more than half of the experimental conditions (as compared with an average of 17%), which suggests either unwillingness or inability to follow the instructions. The resulting sample of 100 adults (15 males, 1 non-binary) ranged in age from 19 to 31 years (*M* = 21.88, *SD* = 2.36). Participants either received course credit or a monetary compensation of 10 € for their participation. The experiment was conducted in accordance with the ethical guidelines of the German Psychological Association (DGPs) and the Professional Association of German Psychologists (Deutsche Gesellschaft für Psychologie, 2016) and with the 1964 Declaration of Helsinki.

### Materials

Four sounds (frog, piano, drum, and bell) with a duration of 300 ms (including on- and off-ramps) were used as target and distractor stimuli. All sounds had an average loudness of approximately 71 dB (A) SPL. NIOSH (2016) on a cellphone (iPhone 8, Apple Inc., Cupertino, USA) equipped with an external microphone (iMM-6 iDevice Calibrated Measurement Microphone, Dayton Audio, Springboro, USA) was used to measure the loudness while the sounds were played on one side of a stereo headphone (DT110, Beyerdynamic GmbH & Co. KG, Heilbronn, Germany). The offline test was programmed by LiveCode (LiveCode 9.5, Runtime Revolution Ltd., Edinburgh, Scotland), whereas the online test was programmed using Psychopy3 (Peirce et al., 2019).

Each presentation started with a 20-ms metronome click either played to the right or left ear. Then, a target sound was played on the side indicated by the metronome click, and a distractor sound was simultaneously played on the other side. Participants were asked to identify the target sound by an appropriate keypress and to ignore the distractor sound. In the offline test, the response keys were “9”, “6”, “3” and “,” on the number pad of the keyboard. These keys were assigned to frog, piano, drum, and bell sounds, respectively. In the online version of the experiment, four common letter keys *F, V, J*, and *N* (assigned to frog, piano, drum, and bell sounds) were selected as response keys to avoid the potential lack of a number pad on privately owned computer systems. All participants were instructed to use the middle and index fingers of their left and right hands to press the keys. Specifically, for the offline test, five participants were instructed to use the middle and index fingers of their right hands to press the two distal keys (i.e., “9” and “6”), and the middle and index fingers of their left hands to press the two proximal keys (i.e., “3” and “,”). This arrangement was reversed for the remaining six participants.

An additional sine tone, presented together with the target and distractor sound pair, served as context. The context tone was played simultaneously to both ears to create the impression to originate from a central location. The context tone could be (1) 150 Hz, (2)300 Hz, (3) 400 Hz, (4) 500 Hz, (5) 600 Hz, (6) 700 Hz, (7) 800 Hz and (8) 900 Hz in frequency. The context tones lasted for 300 ms, including 10-ms attack and decay intervals, and they were approximately equal in loudness as target and distractor sounds. When added to the target and distractor sound pair, the context tones only slightly increased the overall loudness (i.e., within 3 dB(A)).

Twelve context tone pairs were created with the restrictions that each pair only comprised frequencies with even or odd labels (e.g., 300 Hz and 500 Hz or 400 Hz and 800 Hz). With at least 200 Hz difference in frequency, the context tones in a pair could be easily distinguishable from each other. Among these twelve pairs of context tones, the frequencies of occurrence were balanced (i.e., each context tone appeared three times). All twelve pairs of context tones were used in the high-variability group, whereas in the low-variability group, a randomly chosen context sound pair was employed.

Each trial comprised a prime presentation and a probe presentation. Ignored repetition trials were created by selecting three out of the four sounds as the target and distractor in the prime and probe presentations, with the probe target identical to the prime distractor. Replacing the prime distractor with the remaining fourth sound in each ignored repetition trial created a parallel control trial. Note that if only ignored repetition trials and their corresponding control trials were used, participants may learn to expect no response repetition between the prime and the probe. Therefore, attended repetition trials and their parallel control trials were added. The attended repetition trials also required three out of the four sounds to be the target and distractor in the prime and probe presentations, but the restriction was that the probe target was identical to the prime target. Accordingly, the parallel control trials were constructed by replacing the prime target with the remaining fourth sound. Since no hypothesis was made for attended repetition trials and their parallel control trials, their results will not be reported. However, the data were uploaded to PsychArchives for those who are interested.

The basic set of experimental trials contained 48 trials, with 12 trials for each of the four trial types described above.^2^ The basic set was implemented for eight times, four times with the context repeated in the prime and the probe, and four times with the context changed between the prime and the probe. The resulting 384 experimental trials were presented in a random sequence. In each trial, the side where the prime target would be presented was randomly decided, whereas the probe target would always be presented on the opposite side. This was done to avoid identity-location feature mismatches in the ignored repetition condition, which has been discussed as one reason for the emergence of the negative priming effect (Park & Kanwisher, 1994).

### Procedure

**Figure 2:**
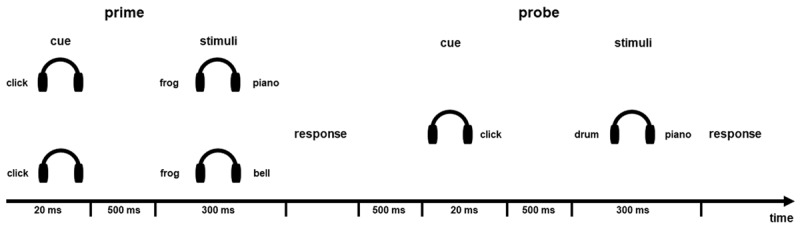
Example of the trial procedure. Primes are shown for the ignored repetition (upper) and control (lower) conditions with an identical probe. *Note*: The contextual stimuli are not presented in the figure. The response interval was 100–3000 ms.

Participants were first informed to use headphones. After being familiarized with the experimental sounds, participants were introduced to the general task. A training phase followed, which included three sessions: In the first session, participant learned about the target and distractor sound pairs without context tones. The task was to identify the target sound by pressing the appropriate key and to ignore the distractor sound. For the offline test, accuracy had to be at least 60% in the 15 subsequent trials to pass this training phase. If the criterion was missed after 60 trials, participants were offered to quit the experiment or to repeat the training. For the online test, the accuracy criterion to pass was set to 33 % in 12 subsequent trials to reduce the overall task duration. If the criterion was missed, participants could either quit the experiment or repeat the training. In the second session, sound pairs were presented together with context tones. Prior to the training session, participants were informed that the context tones were task-irrelevant, and were instructed to focus on the task itself. The criterion to pass the second training session was identical to the first session. Finally, participants responded to six or ten prime and probe trials in the offline or the online version of the experiment, respectively. The timing of the training trials in this session was identical to that of the experimental trials (see ***Figure 2***).

Each experimental trial started with the 20-ms metronome click, which cued the side where the prime target would be presented. Following a 500-ms cue-target interval, the prime sound pair was presented. The prime response was followed by a 500-ms prime-probe interval, after which the probe cue was presented on the opposite side of the prime cue. After another 500-ms interval, the probe sound pair was presented. Participants received audio-visual feedback about the correctness of the prime and the probe responses after each trial. The intertrial interval was set to 1200 ms. Responses faster than 100 ms and slower than 3000 ms were counted as invalid and were excluded from analyses, and participants received warning message about them.

The experiment comprised 16 blocks with 24 experimental trials in each block. After each block, the overall error rate for the block was presented on the screen. Participants could either take a rest or start the next block at their own discretion by pressing one of the response keys (in the offline test) or the space key (in the online test). The testing lasted for approximately 60 minutes, for both online and offline versions.

### Design & Analysis

The experiment was a 2×2×2 mixed design with Trial Type (ignored repetition vs. control), Context Relation (repeated vs. changed) as the within-subject variables, and Context Variability (low vs. high) as the between-subject variable. The dependent variables were averaged reaction times, overall probe error rates, and most importantly, probe response frequencies.

In order to have enough power to observe the basic negative priming effect, sample size was calculated with the purpose to detect a medium-sized effect (i.e., *f* = 0.25, as defined by Cohen, 1988) of context variability on the contextual modulation of the negative priming effect. The calculations were conducted using the G*Power program (Faul et al., 2009). Given desired levels of α = β = .05, and an assumed correlation of ρ = .2 (estimated from Mayr et al., 2018) between the negative priming effects in context repeated and changed conditions, data had to be collected from 86 participants. The final sample comprised 100 participants, so the power was slightly larger (.97) than what was originally planned for. P-values of multiple comparisons were reported after Bonferroni-Holm correction (Holm, 1979).

**Figure 3:**
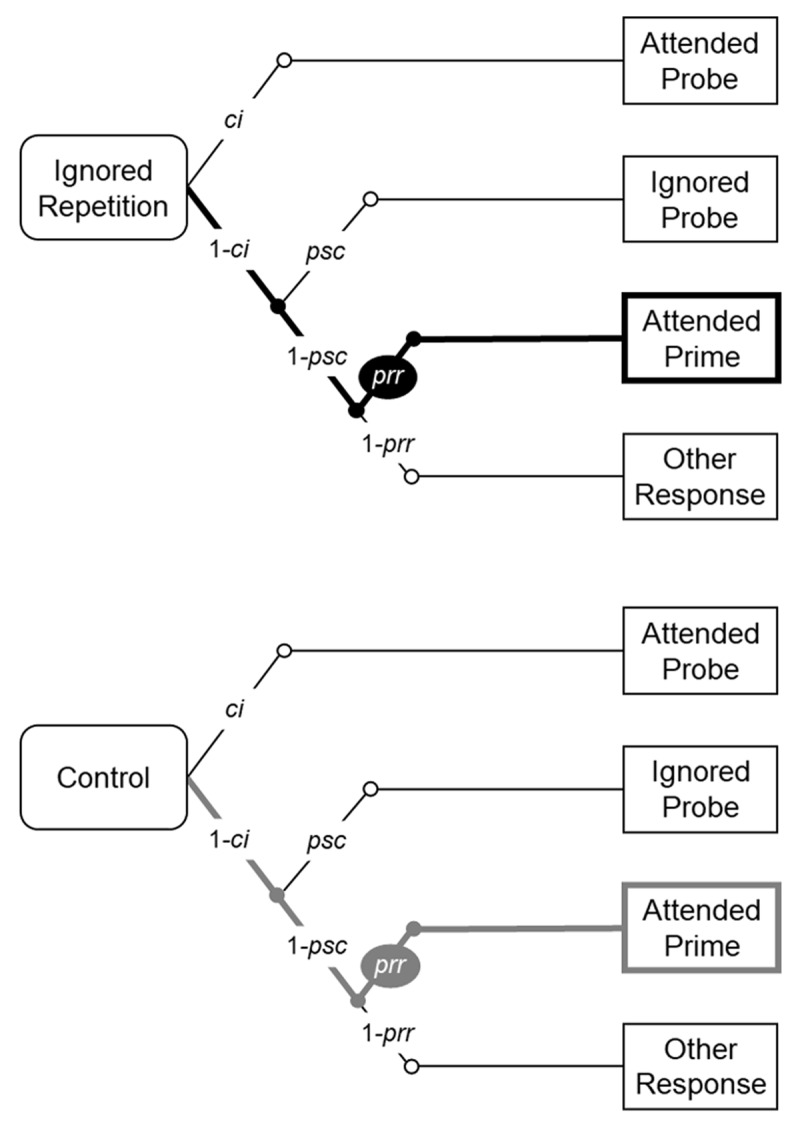
The multinomial processing tree model in the ignored repetition and the control conditions. *Note*: The figure was taken from Qiu et al. (2022).

To estimate the conditional probability of prime response errors based on the probe response frequency data, the multinomial processing tree (MPT) model introduced by Mayr and Buchner (2006) was implemented (see ***Figure 3*** for a description of the model in the ignored repetition and the control conditions). Henceforth, this specific model is referred to as the baseline model. There are four possible probe response categories in the baseline model, namely, (1) the correct response (correct identification, represented by the probability parameter *ci*), (2) incorrect response to the probe distractor (the probe stimulus confusion error, represented by the probability parameter *psc*), (3) incorrect response to the prime target (the prime-response retrieval error, represented by the probability parameter *prr*), and (4) incorrect response with the remaining response option. To investigate the prime-response retrieval effect induced by the repetition of the prime distractor stimulus, a restriction of the equivalence of the *prr* parameters between the ignored repetition condition and the control condition (i.e., *prr*_IR_ = *prr*_C_) is added to the baseline model. A significant misfit between this restricted model and empirical data will be evidence for the prime-response retrieval effect induced by the repetition of the prime distractor stimulus, indicating a binary binding between the prime distractor stimulus and the response.

When testing the integration of the context, the baseline models in the context repeated and changed conditions were integrated into a joint model, for each Context Variability group. The binary binding between the context and the response was tested first, with the restriction of the equivalence of the *prr*_C_ parameters between the context repeated and changed conditions. If the restricted model has to be rejected (i.e., significantly misfits the empirical data), the prime-response retrieval effect induced by the repetition of the context alone is significant, which is evidence for the binary binding between the context and the response.

Finally, the contextual modulation of the binding between the prime distractor stimulus and the response was investigated. The analysis corresponds to an interaction analysis between the Context Relation and Trial Type. The interaction analysis in MPT modeling requires reparameterization of the joint model (see Knapp & Batchelder, 2004 for details of MPT model reparameterization methods; please see the Appendix of Qiu et al., 2022 for detailed description of the reparameterized model and the interaction analysis used in the current study). In the reparametrized model, the prime-response retrieval effect induced by the repetition of the prime distractor stimulus can be represented by the difference of the *prr* parameters between the ignored repetition and the control conditions, that is, *prr*_IR_ – *prr*_C_. If the goodness-of-fit test suggests a significant difference of (*prr*_IR_ – *prr*_C_) between the context repeated and changed conditions, it is assumed to be evidence for the involvement of context in a configural binding, as found in Mayr et al. (2018). The model analysis described above were run with the multiTree software (Moshagen, 2010).

## Results

### Reaction times and error rates

**Figure 4:**
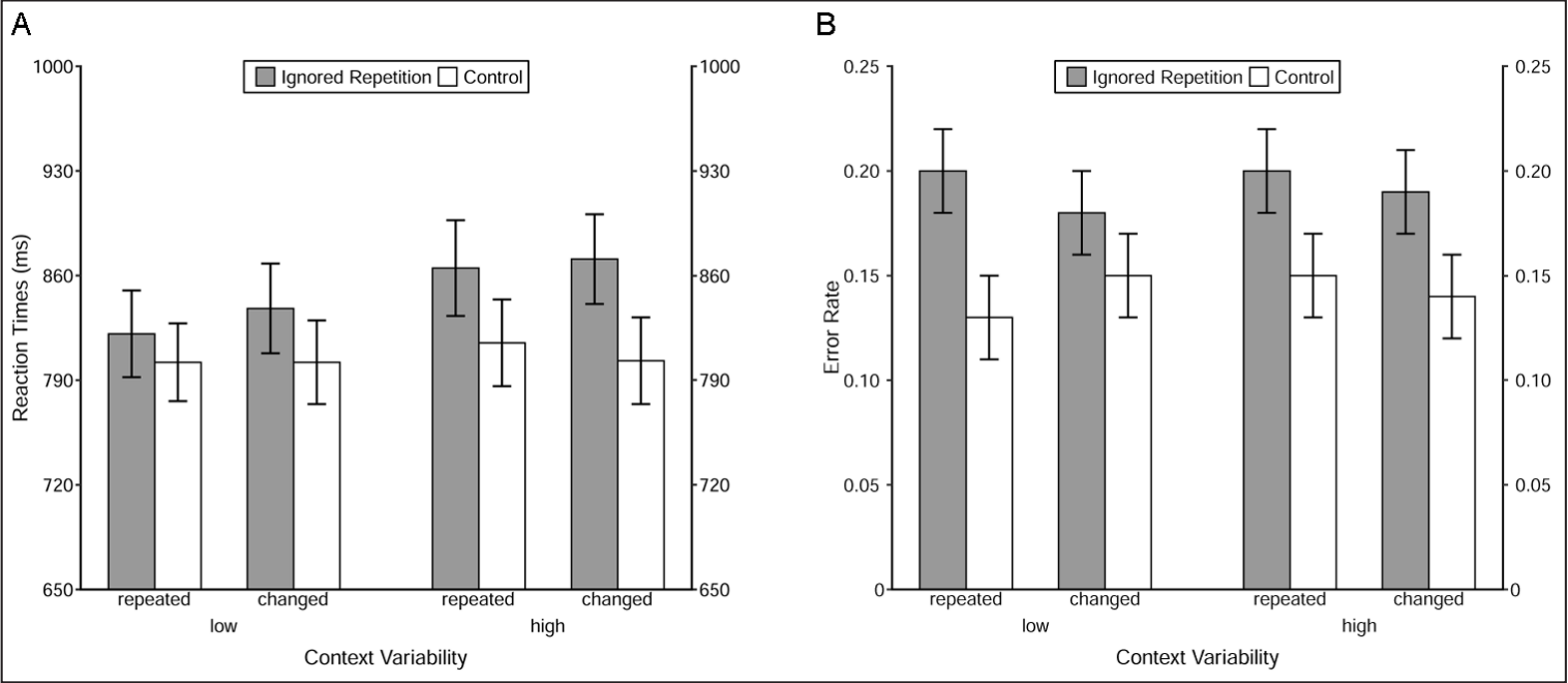
Descriptive findings in reaction times and error rates. *Note*: **A)** Reaction times as a function of Trial Type, Context Relation and Context Variability; **B)** Error rates as a function of Trial Type, Context Relation and Context Variability. The error bars depict the standard errors of the means.

A 2 (Trial Type: ignored repetition vs. control) × 2 (Context Relation: repeated vs. changed) × 2 (Context Variability: low vs. high) mixed models MANOVA was applied to probe reaction times and error rates (see ***Figure 4*** for an overview of descriptive findings). The results showed a significant main effect of Trial Type in reaction times, *F*(1, 98) = 37.54, *p* < .001, η_p_^2^ = .28, and in error rates, *F*(1, 98) = 46.39, *p* < .001, η_p_^2^ = .32. Probe responses were slower and more error prone in ignored repetition than in control trials, revealing a typical negative priming effect (Neill & Valdes, 1992; Tipper, 1985). Moreover, there was a significant interaction between Trial Type and Context Variability in reaction times, *F*(1, 98) = 4.93, *p* = .03, η_p_^2^ = .05. Further analysis showed that the negative priming effect was significant in the high- as well as the low-variability group, but the effect was larger for the former, *F*(1, 49) = 33.42, *p* < .001, η_p_^2^ = .41, than for the latter, *F*(1, 49) = 7.97, *p* = .04, η_p_^2^ = .14. No other interaction or main effects, neither in reaction times nor in error rates, were significant, all *Fs* < 3.01, *ps* > .08.

### MPT model results

**Figure 5:**
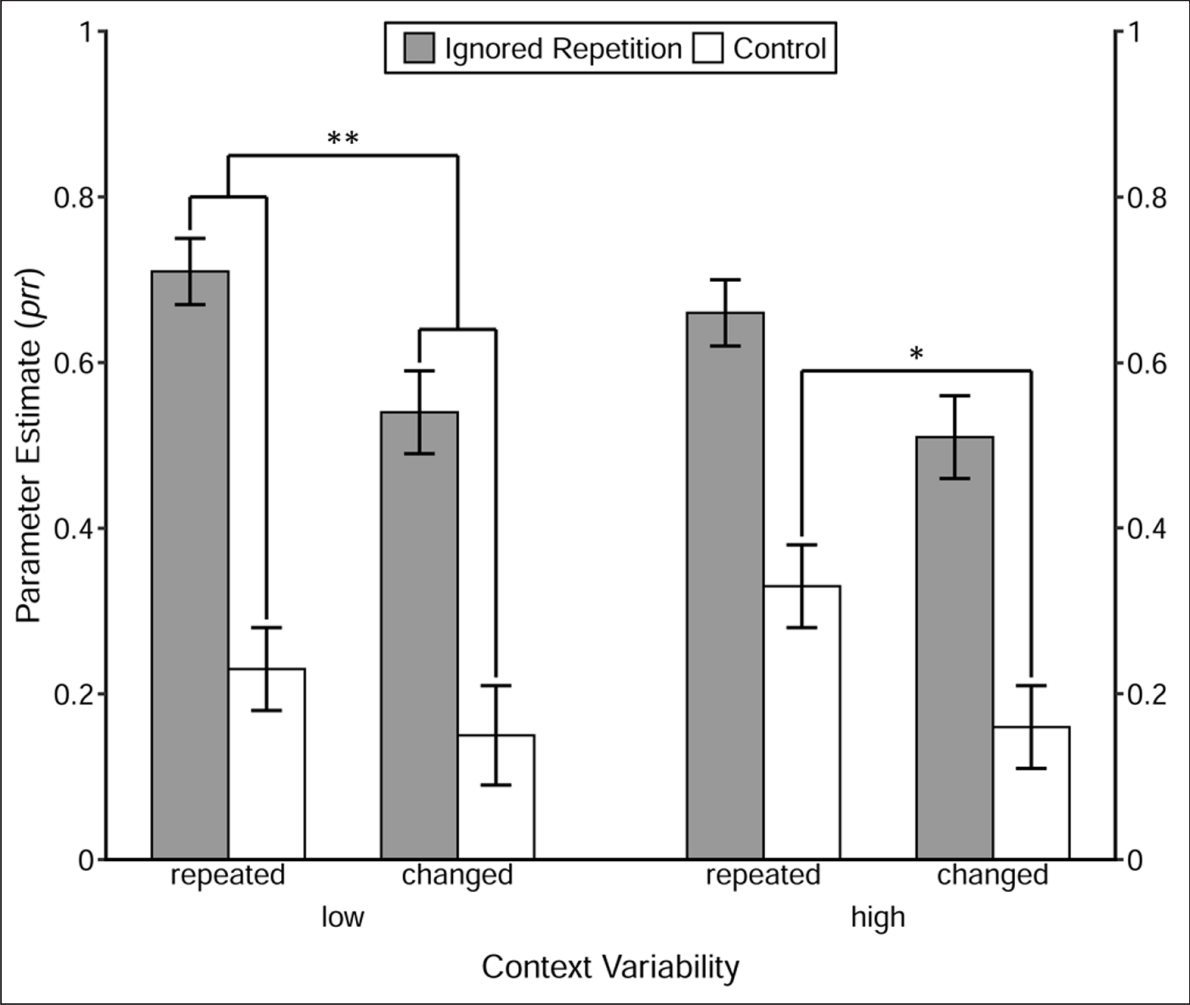
Probability estimates for the model parameters representing the probability of prime-response retrieval (*prr*) as a function of Trial Type, Context Relation and Context Variability. *Note*: The error bars depict the standard errors of the parameter estimates. Annotation shows significant comparisons indicating configural and binary binding of the context. The symbols “**” and “*” indicate *p* < .01 and *p* < .05, respectively.

The estimated value of the *prr* parameters under each 2 × 2 × 2 condition is depicted in ***Figure 5***. The prime-response retrieval effect induced by the repetition of the prime distractor stimulus in each of the 2 (Context Relation) × 2 (Context Variability) conditions was tested first. Results showed that with the restriction *prr*_IR_ = *prr*_C_, the restricted model always had to be rejected, *G^2^s* > 18.95, *ps* < .001, ωs > .06, revealing evidence for the prime-response retrieval process induced by the repetition of the prime distractor stimulus.

Then, the prime-response retrieval effect induced by the repetition of the context per se was tested with the restriction of equivalence of the *prr*_C_ parameters between the Context Relation conditions, in each of the Context Variability groups. The goodness-of-fit test showed that only in the high-variability group, the restricted model had to be rejected, *G^2^*(1) = 5.19, *p* = .02, ω = .03. However, in the low-variability group, the restricted model did not yield a significant misfit, *G^2^*(1) = 1.10, *p* = .29, ω = .01.

Finally, the contextual modulation of the prime-response retrieval effect induced by the repetition of the prime distractor stimulus was investigated using the interaction analysis of MPT modeling. The interaction analysis showed that in the low-variability group, the prime-response retrieval effect induced by the repetition of the prime distractor stimulus was larger when the context was repeated than when the context was changed, *G^2^*(1) = 6.73, *p* < .01, ω = .03. However, no significant contextual modulation was found in the high-variability group, *G^2^*(1) = 0.09, *p* = .77, ω < .01.

## Discussion

In the current study, the influence of stimulus inter-trial variability on the binding structure of the context in an S-R episode was investigated. The variability property of context was manipulated by using either two different sine tones or eight different sine tones as the context. In the former condition which equals the study by Mayr et al. (2018) with respect to the composition of the context stimuli, results replicated the previous finding that the sole repetition of the context did not lead to a significant prime-response retrieval effect. Also in line with Mayr et al. (2018), a larger prime-response retrieval effect induced by the repetition of the prime distractor stimulus was found when the context was additionally repeated than when the context was changed. In the high-variability condition of the context, however, results showed that the repetition of the context per se induced a significant prime-response retrieval effect, which is evidence for the binary binding between the context and the response. Together, the current study reveals that the inter-trial variability is a determinant of the binding structure of context in an S-R episode.

The current results are in line with the attention hypothesis proposed by Chao (2009). Assuming that increasing the variability of the context leads to an increase of attention allocated to the context stimulus during processing, in the high-variability group, the presumably better encoded context stimulus may be more strongly/likely integrated into an episodic representation directly with the response, resulting in a binary binding structure. In the low-variability group, on the other hand, with less attention during processing, the context may be less discriminable from other stimuli in the prime episode, thereby entering into a configural binding with the prime distractor stimulus and the response (Moeller et al., 2016). This combined pattern of results–binary binding in the high-variability group and configural binding in the low-variability group–presumably is due to the fact that the manipulation of the inter-trial variability in the current study influenced the perceived novelty of the context stimulus. The more frequently changing context may be perceived as more novel, thereby attracting more attention (e.g., Ernst et al., 2020; Parmentier, 2008). Thus, increasing the variability can lead to a binary binding between the context and the response, as shown in the current study.

Note that the current results also reveal that the context is integrated into an S-R episode regardless of its inter-trial variability. Assuming the manipulation of the inter-trial variability influenced attention allocated to the contextual stimulus, the current results are in line with previous findings that attention does not determine whether a stimulus is integrated into an S-R episode or not (e.g., Giesen et al., 2012; Hommel, 2005; Hommel & Colzato, 2004; Moeller & Frings, 2014). However, the current result patterns (i.e., binary binding in the high-variability group and configural binding in the low-variability group) further extend these previous findings, as it suggests that attention may influence the specific structure of the binding that a stimulus is involved in. Presumably, with more attention, the stimulus is more likely to be bound in a binary fashion with the response, otherwise it may enter into some kind of a higher-order binding with other stimuli and the response.

Another possible reason for the current result patterns comes from the *figure-ground segmentation* literature (for a review, see Wagemans et al., 2012).^3^ It was found that the so-called “figural” stimulus presented in a confined area on the screen entered into a binary binding with the response, whereas the “ground” which covered the whole screen did not (Frings & Rothermund, 2017). Transferring the “figure” and the “ground” into the auditory modality, the former may be in analogy with an individual auditory object, whereas the latter may be in analogy with a background sound of other stimuli. If the manipulation of the inter-trial variability influences whether the context is perceived as an individual object or a background of other stimuli, then the context of high variability may more likely be perceived as an individual object, thereby entering into the binary binding with the response (Frings & Rothermund, 2017). The context of low variability, on the other hand, may more likely be perceived as the background of other stimuli. Therefore, it forms a “compound” with the other stimuli and enters into the configural binding with the prime distractor stimulus and the response (see Qiu et al., 2022 for a similar explanation). With that being said, whether and how the inter-trial variability influences the figure-ground perception of the contextual stimulus requires further investigation in future studies.

Finally, in the current study, the Context Relation factor did not affect the negative priming effect, neither in reaction times nor in error rates (note that some previous studies also reported insignificant influence of Context Relation on the negative priming effect, e.g., Eben et al., 2020; Wong, 2000). Instead, an influence of Context Variability was found on the negative priming effect, with a larger negative priming effect when the context variability was high than when it was low. This result is different from what was found by Chao (2009), which showed a significant negative priming effect only when the context was repeated in the high-variability condition. However, the current result is consistent with the previous finding that the prime-response retrieval process is the only mechanism underlying the negative priming effect that is sensitive to the contextual modulation in the auditory modality (Mayr et al., 2018). Prospective studies are required to investigate whether and how the difference in modality influences the role of context in negative priming.

To sum up, the current study investigated the binding structure of the context in an S-R episode by manipulating the inter-trial variability of context. Results show that with high variability, the context enters into a binary binding with the response; whereas with low variability, the context is involved in a so-called configural binding with the distractor stimulus and the response. Together, the current study indicates that the inter-trial variability of context is a determinant of its binding structure in an S-R episode, thereby providing insights into the influence of contextual information on action control.

## Data Accessibility Statement

The data and programming code for data analysis of the experiment are available on *https://doi.org/10.23668/psycharchives.5605*. The experiment was not preregistered. This work is part of the doctoral dissertation by Ruyi Qiu.

## Footnotes

Note that there are several possible processes underlying the negative priming effect (for a review, see Frings et al., 2015). Apart from the memory-based processes (e.g., Neil & Valdes, 1992) which are mainly focused on in the current paper, an inhibition mechanism has also been discussed as a reason for the emergence of the effect (Tipper, 1985).

Note that the full permutation of the four sounds actually leads to 24 trials for each trial type, resulting in a complete set of 96 trials. However, in this set of 96 trials, the ignored repetition trials and the attended repetition trials always share an identical set of control trials, which means that each control trial would have occurred twice. Therefore, the ignored repetition and attended repetition trials were systematically assigned to two basic sets, with the restriction that none of the control trial was repeated in one set. It was randomly decided which basic set each participant was assigned to. Please see Mayr and Buchner (2006) for more details about the basic sets.

We thank the anonymous reviewer for this possible alternative account.
